# Using Best-Worst Scaling to assess preferences for online psychological interventions to decrease cannabis use in young adults with psychosis

**DOI:** 10.1192/j.eurpsy.2023.1119

**Published:** 2023-07-19

**Authors:** O. Tatar, A. Abdel-Baki, H. Bakouni, A. Bahremand, T. Lecomte, J. Côté, D. Crockford, S. L’Heureux, C. Ouellet-Plamondon, M.-A. Roy, P. G. Tibbo, M. Villeneuve, D. Jutras-Aswad

**Affiliations:** 1 Department of Psychiatry and Addiction; 2 Research Center, Centre Hospitalier de l’Université de Montréal (CRCHUM); 3 Department of Psychiatry and Addicttion; 4 Department of Psychiatry, Centre Hospitalier de l’Université de Montréal (CHUM); 5 Department of Psychology; 6 Centre de Recherche de l’Institut Universitaire en Santé Mentale de Montréal; 7 Faculty of Nursing, Université de Montréal, Montreal; 8 Department of Psychiatry, University of Calgary, Calgary; 9 Clinique Notre-Dame des Victoires, Institut universitaire en santé mentale de Québec (IUSMQ); 10 Département de Psychiatrie et Neurosciences, Laval University, Québec; 11 Department of Psychiatry; 12 Nova Scotia Early Psychosis Program, Dalhousie University, Halifax, Canada

## Abstract

**Introduction:**

In individuals with first episode psychosis (FEP) and cannabis use disorder (CUD), reducing cannabis use is associated with improved clinical outcomes. Access to evidence-based psychological interventions to decrease cannabis use in FEP clinics is highly variable; E-mental health interventions may help to address this gap. Development of E-interventions for CUD in individuals with FEP is in its incipient phases.

**Objectives:**

To assess preferences for online psychological interventions aiming at decreasing or stopping cannabis use in young adults with psychosis and CUD.

**Methods:**

Individuals aged 18 to 35 years old with psychosis and CUD were recruited from seven FEP intervention programs in Canada and responded to an electronic survey between January 2020-July 2022. We used the Case 2 Best Worst Scaling methodology that is grounded in the trade-off utility concept to collect and analyse data. Participants selected the best or worst option for each of the nine questions corresponding to three distinct domains. For each domain we used conditional logistic regression and marginal models (i.e., three models in total) to estimate preferences for attributes (e.g., duration, frequency of online intervention sessions) and attribute levels (e.g., 15 minutes, every day).

**Results:**

Participants (N=104) showed higher preferences for the following attributes: duration of online sessions; mode of receiving the intervention; method of feedback delivery and the frequency of feedback from clinicians (Table 1). Attribute-level analyses showed higher preferences for participating once a week in short (15 minutes) online interventions (Figure 1). Participants valued the autonomy offered by online interventions which aligns with their preference for completing the intervention outside the clinic and only require assistance once a week (Figure 2). Participants’ preferences were higher for receiving feedback related to cannabis consumption both from the application and clinicians at a frequency of once a week from clinicians (Figure 3).

**Table 1.** Preferences for Attributes. Results of conditional logistic regression

**Attributes**	**Domains**	**OR**	**95% CI for OR**
Duration session	A	**1.62**	**1.45; 1.82**
Frequency sessions		0.98	0.87; 1.09
Duration intervention		ref	
Preferred mode of receiving the intervention	B	**1.63**	**1.46; 1.83**
Preferred location for participating		1.07	0.96; 1.20
Frequency of assistance from the clinician		ref	
Preference for the feedback delivery method	C	**1.21**	**1.08; 1.36**
Frequency of feedback from the treating clinician		**1.14**	**1.02; 1.28**
Frequency of feedback from the application		ref	

Note: In boldface significant odds ratios (OR) and confidence intervals (CI)

**Image:**

**Figure:**
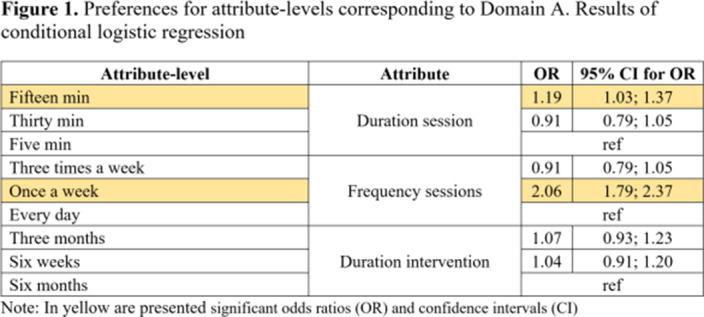

**Image 2:**

**Figure:**
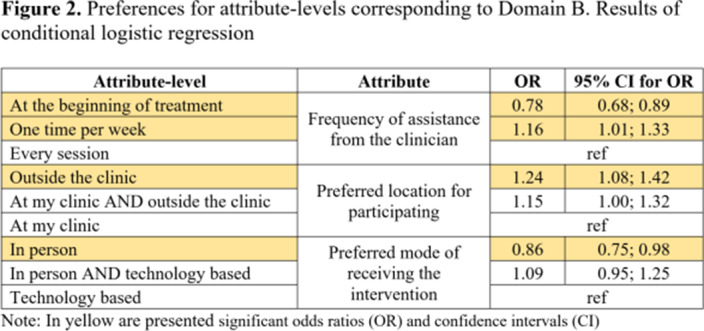

**Image 3:**

**Figure:**
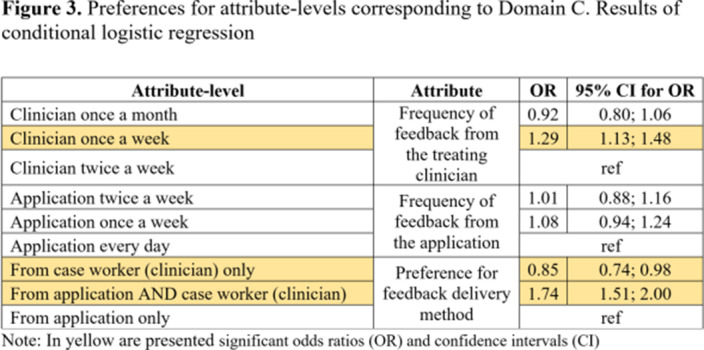

**Conclusions:**

Using advanced methodologies to assess preferences, our results can inform the development of highly acceptable E-Mental health interventions for decreasing cannabis use in individuals with CUD and FEP.

**Disclosure of Interest:**

None Declared

